# Comparing Participation and Interim Effectiveness of Endoscopy and Biomarker-Based Screening for Gastric Cancer: A Cluster Randomized Controlled Trial

**DOI:** 10.7150/jca.99100

**Published:** 2024-10-07

**Authors:** Haifan Xiao, Hao Luo, Ang Qin, Wenxian Shu, Xiangyu Liu, Fengfan Xiao, Xianzhen Liao, Zhaohui Shi, Yanhua Zou, Kekui Xu, Shiyu Cao, Can Li, Yingyun Hu, Senmao Zhang, Jia Guo, Shiyu Wang, Shipeng Yan

**Affiliations:** 1 The Department of Cancer Prevention and Control, Hunan Cancer Hospital/The Affiliated Cancer Hospital of Xiangya School of Medicine, Central South University/Hunan Cancer Prevention and Treatment Center, 283 Tongzipo Road, Changsha 410013, China.; 2 Xiangtan Center for Disease Control and Prevention, No. 12 North Second Ring Road, Economic& Technology Development Zone, Xiangtan 411100, China.; 3 The Department of Endoscopic Centre/Ambulatory Surgery Center, Hunan Cancer Hospital/The Affiliated Cancer Hospital of Xiangya School of Medicine, Central South University, 283 Tongzipo Road, Changsha 410013, China.; 4 The Second Department of Thoracic Surgery, Hunan Cancer Hospital/The Affiliated Cancer Hospital of Xiangya School of Medicine, Central South University, 283 Tongzipo Road, Changsha 410013, China.; 5 The Department of Health Service Center, Hunan Cancer Hospital/The Affiliated Cancer Hospital of Xiangya School of Medicine, Central South University, 283 Tongzipo Road, Changsha 410013, China.; 6 The Department of Breast medicine, Hunan Cancer Hospital/The Affiliated Cancer Hospital of Xiangya School of Medicine, Central South University, 283 Tongzipo Road, Changsha 410013, China.

**Keywords:** Endoscopic screening, Biomarker detection, Participation, Effectiveness, Gastric cancer

## Abstract

**Background:** To improve compliance with endoscopic screening for gastric cancer (GC), we assessed five biomarkers—pepsinogen I (PG I), pepsinogen II (PG II), PG I/II ratio, helicobacter pylori antibody (HP-Ab), and gastrin 17 (G17) — for secondary GC screening by comparing participation and effectiveness of traditional endoscopy and biomarker-based screening in a randomized trial with baseline results.

**Methods:** Seventy-four communities were randomly assigned to traditional endoscopy arm (TEA) or biomarker-based endoscopy arm (BEA). TEA uses a questionnaire for risk assessment, and BEA combines a questionnaire with biomarker detection. High-risk individuals in both arms underwent endoscopic screening. Participation and interim screening effectiveness in two arms were reported with baseline analysis.

**Results:** In total, 5,798 participants in TEA and 5,158 in BEA were recruited, with a participation rate of 26.9%. BEA showed a significantly lower high-risk rate than TEA (15.2% vs. 38.9%) and a higher endoscopic participation rate for high-risk individuals (64.9% vs. 53.0%). The endoscopic screening results showed that there was no significant difference in detection rate of GC abnormalities between the two arms. Education level, frequent drinking, hot, rough and hard food consumption, family history of GC, and history of reflux esophagitis or gastropathy influenced participation rates in biomarker-based screening. Age group, sex and regular consumption of meat, eggs and milk products were associated with stomach abnormalities.Cumulative incidence and specific death rates did not significantly differ in intention-to-screen and per-protocol analyses.

**Conclusions:** Biomarker-based screening effectively identifies high-risk individuals and increases endoscopic participation, providing value insights for improving screening efficiency as a secondary procedure.

## Introduction

The incidence of gastric cancer (GC) is increasing globally 1. In China, the incidence and mortality rates of GC ranked fifth and third highest among all cancers, with 0.36 million new cases and 0.26 million deaths 2. Early detection and treatment of GC are crucial for improving survival rates and reducing disease-specific mortality 3. Endoscopy has been widely used as the gold standard for GC screening, and previous studies in high-incidence Asian countries, including China, Japan, and Korea, have demonstrated its effectiveness in reducing incidence and mortality rates^3^-^6^. However, traditional endoscopic screening has limitations, such as invasiveness, low compared and higher cost 7. In response to these limitations, biomarker-based endoscopic screening has attracted attention as a more comfortable and less invasive alternative^8^. In recent years, serum pepsinogen (PG) I, PGII, and gastrin-17 (G17) levels have been developed as atrophic gastritis biomarkers 9-12. Furthermore, anti-*Helicobacter pylori* immunoglobulin (Ig) G antibody has been used to detect *H. pylori* infection 9-12. In China, a panel of five biomarkers (serum PGI, PGII, PGI/II ratio, G17, and anti-*H. pylori* IgG antibody) was used to evaluate the high-risk population for GC; this model showed better prescreening performance^11^. Another Chinese nationwide multicenter study reported that the PGI/II ratio, G-17 level, *H. pylori* infection, and other demographic variables could primarily identify high-risk individuals^9^. However, no randomized trials in China have evaluated the suitability of these biomarkers for secondary screening before endoscopy.

To address this gap, our study aimed to conduct a cluster randomized controlled trial comparing the participation and effectiveness of traditional endoscopic and biomarker-based screening methods in a community-based population. Our goal was to explore the validity and feasibility of this novel screening approach, providing evidence-based medicine for identifying, classifying, screening, and intervening in high-risk individuals with GC. This mid-term analysis focused on analyzing participation and preliminary screening effectiveness in the target population, with the aim of establishing a scientifically sound and contextually suitable screening strategy.

## Materials and methods

### Study design

This study commenced on March 1, 2019, and recruitment and baseline screening were completed by December 31, 2019. We employed a cluster randomized controlled trial design, similar to our previous study^13^, and aimed to include 10,000 participants as explore study in Hunan Province where was considered as non-high risk area. Our research received approval from the Ethics Committee of the Cancer Hospital, Chinese Academy of Medical Sciences (Approval No. 18-179/1757). The research was carried out in adherence to the principles outlined in the Declaration of Helsinki.

This study was performed in Changsha and Xiangtan city, from which reliable tumor and death surveillance data could be obtained. In addition, they had good foundation of cancer screening as the major national public health project -- Early Screening Program in Urban China (CanSPUC) was implemented in those cities. In total, there were 74 communities in those two cities, which were served as cluster units and were randomly assigned to the traditional endoscopy arm (TEA) and biomarker-based endoscopy arm (BEA) at a 1:1 ratio using random numbers generated from the function “rand()” in Microsoft Excel 2013.

### Subject enrollment

All subject recruitment in the study was completed in the community by general practitioner (GP). In our study, GPs need meet the following criteria: medical education background with a college degree or above, 3 years of experience in community work, and participation in the recruitment skills training organized by the project team. Community doctors recruited subject based on two ways. Active Enrollment: The local district center for disease control and prevention (CDC) or community will actively promote the project to encourage community residents to voluntarily register at the community and be seen by the assigned GP. Passive Enrollment: Each community holds a list of age-eligible residents (40-69 years old) in its jurisdiction. GPs will check out the list by phone or home visits, and invite residents who are interested in participating in the project. Then they will make an appointment to the community for registration. All registration participants were provided written informed consent.

The inclusion criteria were in accordance with our previous study 13: Participants must be local residents aged 40-69, without any history of cancer, and have not undergone endoscopic examination in the past 3 years. Additionally, they must be in good mental and physical condition. Participants were excluded if they were unwilling to participate and had undergone endoscopic screening within the previous three years.

### Screening procedures

**Figure 1:**
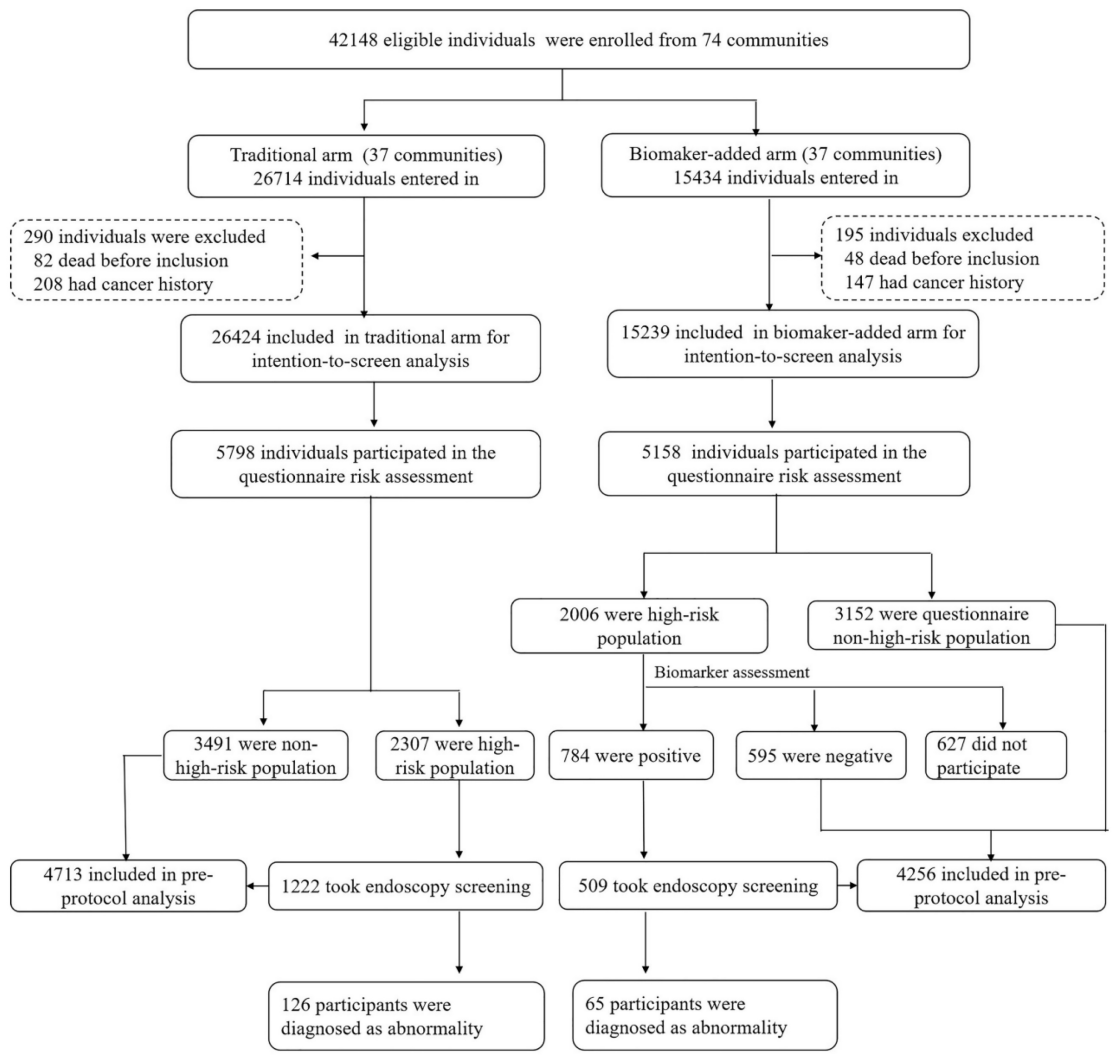
Baseline screening flow diagram of our study.

In the TEA, participants identified as high risk for GC through the risk factor questionnaire assessment underwent endoscopy as the intervention measure. However, the BEA was added to biomarker tests after the questionnaire assessment and before endoscopy (Figure 1).

#### High-risk evaluation

Because endoscopic screening can screen esophagus and stomach at the same time, this study conducted opportunistic screening for esophageal cancer at the same time. Therefore, both esophageal cancer (EC) and gastric cancer were evaluated in the high-risk assessment questionnaire.

An epidemiological questionnaire was administered to all participants enrolled in the study. As our previous study reported 13, the questionnaire encompasses eight sections: demographics, behavioral habits, food frequency survey, personality and mental health, medical history, family history of cancer, clinical symptoms of EC and GC, and physical examination. Participants were considered to be at high risk for GC if they exhibited any two items from the following ①-④, or any one item from ⑤-⑧: ① regular smoking (20 cigarettes/day for over 10 years), ② regular drinking (50g of white wine/day for over 10 years, the alcohol content is usually 34 to 53 percent), ③ frequent consumption of mildewed, fermented and high-salt containing food, ④ consumption of hot, rough and hard foods, Such as hard pancakes, fried shrimp and so on, ⑤ a family history of GC, ⑥clinical symptoms of EC (e.g., retrosternal or subxiphoid pain while eating, progressive dysphagia), ⑦ clinical symptoms of GC (e.g., loss of appetite, abdominal distension, heartburn and regurgitation, malignant vomiting, belching, hematemesis, black stool, progressive wasting, etc.), ⑧ and a history of reflux esophagitis or gastropathy.

#### Biomarker detection

Biomarker detection included human PGI, human PGII, human *H. pylori* antibody (HP-Ab), and human G17. An enzyme-linked immunosorbent assay kit from Jiangsu Meimian Industrial Co., Ltd in China 14, employing the double-antigen sandwich method, was used. Results were considered positive if any of the following criteria were not within the specified range: ① PGI/PGII=0.1-0.45 (PGⅠ=6.38-42.53 µg/L, PGII=56.53-377.25 µg/L) µg/L; ② HP-Ab<422 ng/L; or ③ G17<545 pg/ml.

#### Endoscopic screening

Participants identified as high-risk in both arms underwent endoscopic screening at a designated hospital. In the BEA, participants underwent a combined and sequential risk assessment with firstly a questionnaire and then biomarker detection. Patients were considered high-risk if both questionnaire and biomarker tests yielded positive results. In the study, endoscopists were blinded to the patient groups (TEA vs BEA) or the results of serum biomarkers. Moreover, there were four endoscopists with at least 5 years of experience in endoscopy in both the Changsha and Xiangtan project areas. The Olympus endoscope GIF-H260 was used. During the endoscopic procedure, if any suspicious lesions are detected, Indigo Carmine staining is employed to enhance visualization. A biopsy is then precisely performed on the areas exhibiting abnormal staining.

### Follow-up, re-examination and outcomes

We followed up all enrolled participants every 12 months, utilizing both active and passive methods. The final follow-up was completed on May 31, 2023.

Once a positive result is detected, patients are promptly notified to undergo re-examinations, adhering to the identical technique employed during the initial baseline screening. The re-examination schedule is as follows: individuals with mild esophageal dysplasia are required to revisit for a recheck every three years, while those with moderate esophageal dyspepsia, cardiac or gastric low-grade intraepithelial neoplasia, severe atrophic gastritis, or severe intestinal metaplasia must undergo annual re-examination.

Primary outcomes comprised GC incidence rate and mortality caused by GC. Participants who tested positive during endoscopic screening underwent active follow-up through telephone or in-home visits. In contrast, individuals who tested negative were passively followed up through provincial linkages with cancer registration and death monitoring databases, ensuring reliable accuracy. Clinical staging was based on the AJCC TNM Staging of Gastric Cancer (8th Edition).

### Quality control

#### Data quality control

The epidemiological investigation, screening procedure, and follow-up data collection stages are subject to three levels of quality control. This encompasses investigator self-verification, periodic data verification by quality controllers, and logical data verification by information systems.

#### Loss of follow-up control

Baseline investigation stage: Collect contact information of the enrolled subjects through multiple channels, including relatives, friends, work units, and neighborhood committee telephone numbers.

Follow-up stage: Loss to follow-up typically includes cases of loss of contact, relocation, or refusal to participate in visits. 1) For individuals lost to contact or relocated, outcomes were obtained by querying the national chronic disease system; 2) For those refusing visits, a sequential sampling or household survey approach was used; 3) Cases with incomplete diagnosis and treatment information found during follow-up were referred by the provincial cancer prevention and treatment center to medical institutions to obtain detailed diagnosis and treatment information.

### Statistical analysis

Means and standard deviations are employed to describe continuous variables. The Student's t-test was used for statistical analysis if a normal distribution was confirmed in the Shapiro-Wilk test and a rank-sum test was used if not (according to the Mann-Whitney U test). Descriptive statistics for categorical variables include frequencies and percentages, and the *χ^2^* test (or Fisher's exact test) was applied for statistical analysis. To identify independent risk factors for screening participation in both groups, logistic regression analysis was performed using a backward step-down process and the likelihood ratio test. We conducted per-protocol analyses to compare participation of endoscopy for two arms, and intention-to-screen and per-protocol analyses to estimate the screening effect. This study included all eligible individuals in 74 communities in an intention-to-screen analysis. Furthermore, all individuals who adhered to the study protocol were included in per-protocol analyses. These included individuals who were not in the questionnaire high-risk population, whose biomarker test was negative, and who underwent endoscopic screening. However, those who did not participate in the high-risk evaluation and did not undergo endoscopic screening, though in the high-risk population, were excluded from the per-protocol analyses. To calculate the hazard ratio (HR) in both study groups, univariate Cox regression analysis was utilized.

Data management and statistical analyses were conducted using Microsoft Excel 2013 and SPSS version 25.0 (IBM Corp., Armonk, NY, USA). Two-sided statistical tests were employed, with a significance level set at P<0.05 to determine statistical significance.

## Results

### Baseline recruitment and high-risk rate of questionnaire risk assessment

In total, 60 communities in Changsha and 14 in Xiangtan were included with 37 communities randomized to the TEA group and 37 communities to the BEA group. A total of 42,148 eligible individuals participated, with 26,714 in the TEA group and 15,434 in the BEA group. After excluding participants with a history of cancer or pre-existing mortality, 26,424 individuals remained in the TEA group and 15,239 in the BEA group. In total, 5,798 individuals in the TEA and 5,158 in the BEA groups participated in the questionnaire risk assessment. The detailed flow chart is presented in Figure 1. The TEA and BEA comprised 2,418 (41.7%) and 1,983 (38.4%) men, respectively. The mean ages in the two arms were similar (57.97 *vs.* 57.80 years, *P*>0.05). Other demographic variables were compared between the two arms (Table 1). In total, 2,006 (38.9%) and 2,307 individuals (39.8%) were estimated to be at high risk in the TEA and BEA, respectively. We also compared the high-risk rates between the two arms based on demographic variables (Table 1).

### Biomarker assessment

In the TEA group, 1,222 high-risk populations participated in the endoscopic screening for GC, yielding a participation rate of 53.0%. In the BEA, 1,379 among 2,006 high-risk participants, based on the questionnaire, further underwent biomarker detection, among which 784 individuals' results were positive. Among them, the positive number of biomarker detection with PGI/PGII ratio, HP-Ab and G17 were 356 (45.4%), 412 (52.6%) and 268 (34.1%) participants, respectively. Therefore, the combined high-risk rate of the questionnaire and biomarker detection in the biomarker-based screening arm was 15.2%, which was lower than that (38.9%) in the TEA based on questionnaire evaluation alone (*P*<0.001). Furthermore, 509 combined high-risk individuals in the BEA underwent endoscopic screening, with a participation rate of 64.9%.

### Endoscopic screening

Endoscopic screening results showed 126 participants (10.2%) in the TEA group and 65 (12.7%) in the BEA group were pathologically diagnosed with GC abnormalities with no significant difference in detection rate (*P*=0.145). To be specific, one of intramucosal adenocarcinoma, 73 of atrophic gastritis, 32 of mild dysplasia, 14 of uncertain dysplasia, and 6 of intestinal metaplasia were detected in TEA group. In the BEA group, we found 1 of intramucosal adenocarcinoma, 31 of atrophic gastritis, 30 of mild dysplasia, and 3 of intestinal metaplasia. Among 65 gastric abnormalities participants, the sensitivity of single biomarker with PGI/PGII ratio, HP-Ab and G17 were 41.5%, 52.3% and 38.5%, respectively, with no significant difference in detection rate (*P*=0.197). The combination of two markers, in which HP-Ab and PGI/PGII ratio in parallel has the highest sensitivity with 87.7%, followed by the combination of PGI/PGII ratio and G17 with 72.3% and HP-Ab as well as G17 with 69.2%.

Furthermore, 36 participants (2.0%) had abnormal esophageal lesions in two groups, including 19 cases of esophagitis, 12 of basal cell hyperplasia, 2 of uncertain dysplasia, 2 of mild dysplasia, and 1 of severe dysplasia.

We also analyze the influencing factor on gastric abnormalities. Through Logistic multivariate analysis age group (OR=1.30, 95%CI=1.09-1.51, *P*=0.014), Female sex (OR=0.65, 95%CI=0.35-0.95, *P*=0.006) and regular consumption of meat, eggs and milk products (OR=0.60, 95%CI=0.28-0.92, *P*=0.002) were found associated with stomach abnormalities.

### Screening compliance in the two arms

The BEA participation rate exceeded that of the TEA (64.9% *vs.* 53.0%; *P*<0.01). A univariate analysis was carried out to determine the endoscopy participation rate in both arms, considering the baseline characteristics and potential risk factors for GC (Table 2). Additionally, a subgroup analysis was conducted to compare the adherence to screening between the two study groups based on baseline characteristics and potential risk factors for GC. The findings indicated that most of the parameters of the participation rate were different between the two arms, except for the 50-59-year age group and having a family history of GC, dysphagia, swallowing pain, black stool, and progressive emaciation symptoms (*P*>0.05, Table 2).

### Factors influencing screening compliance

We utilized multivariate logistic regression analysis to identify the factors impacting screening adherence in the two study arms. For the TEA, older age middle household income (40,000-80,000 Yuan/year), or having dyspepsia, digestive tract symptom and progressive emaciation were associated with a higher endoscopy screening compliance. Meanwhile, frequent smoking was associated with a lower participation rate.

**Figure 2:**
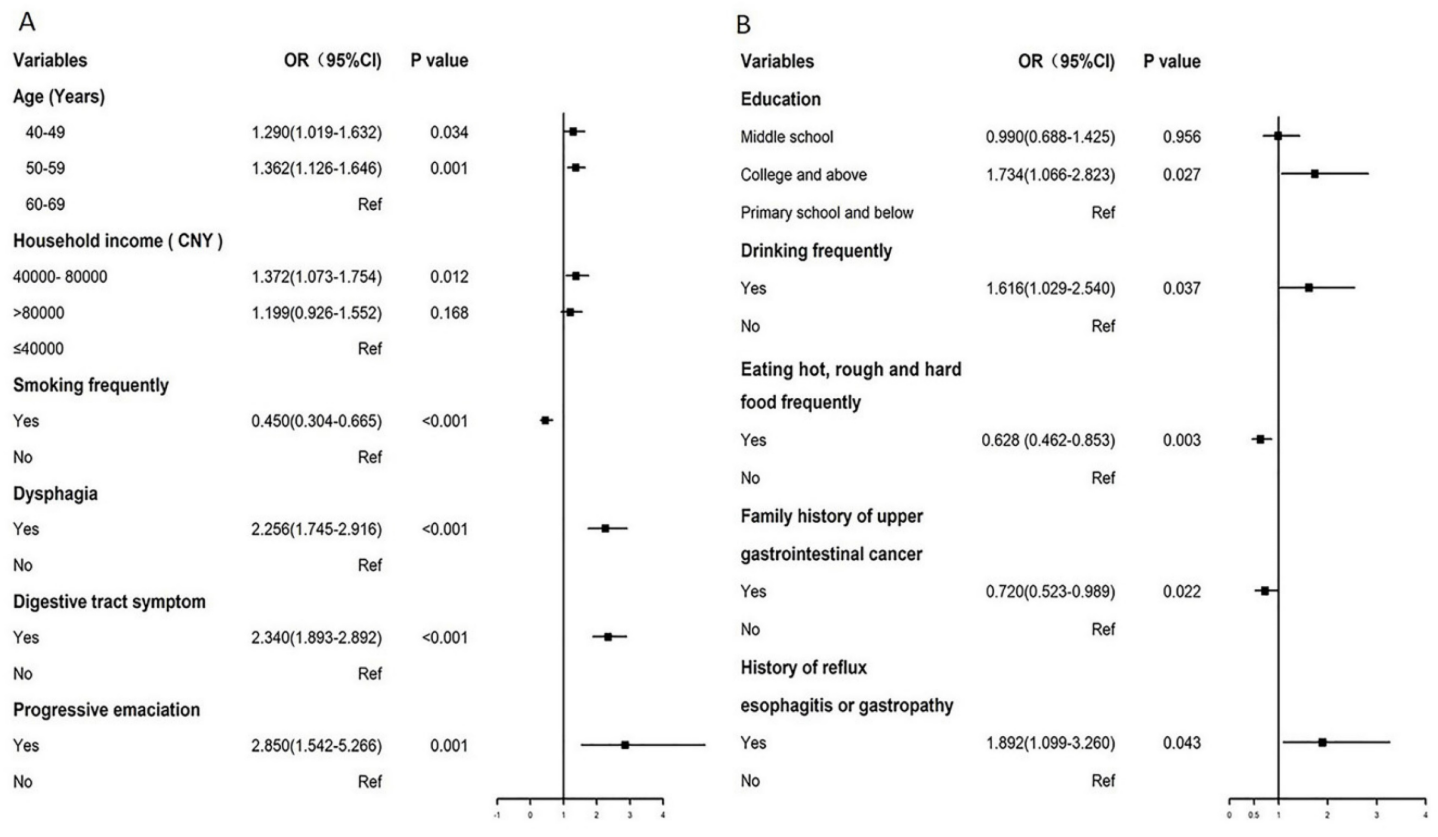
Forest plot of influence factors in biomarker-based screening (A) and traditional endoscopy screening (B).

For the BEA, high household income (>80,000 Yuan/year), family size (<3), family history of GC, and digestive tract symptoms were associated with higher biomarker detection compliance. Individuals who tested positive for the biomarkers were advised to undergo endoscopy. The results revealed that college and higher education, frequent drinking, and history of reflux esophagitis or gastropathy were related with higher endoscopy screening compliance, whereas frequent consumption of hot, rough and hard food and family history of GC were associated with lower endoscopy compliance (Table 3, Figure 2).

### Screening effectiveness evaluation

In the intention-to-screen analysis, 26,424 individuals in the TEA and 15,239 in the BEA were included in the intention-to-screen data analysis. After follow-up (median follow-up duration: 3.90 years; interquartile range: 3.81-3.93 years), 22 and 24 were diagnosed with GC in the BEA and TEA groups, respectively. The cumulative incidences were not significantly different (OR=1.99, 95% CI=0.96-4.13, *P*=0.066). Additionally, no significant variation was observed between the two groups regarding specific deaths attributed to GC (n=7_ BEA_ *vs.* 4_ TEA,_ OR=3.03, 95% CI=0.87-10.64, *P*=0.083) (Table 4).

The pre-protocol analyses included 4,713 individuals in the TEA and 4,256 in the BEA. After follow-up (median follow-up duration: 3.56 years; interquartile range: 3.33-3.75 years), the cumulative incidences were not significantly different (n=6 _BEA_ *vs.* 3 _TEA,_ OR=2.29, 95% CI=0.57-9.17, *P*=0.241). Only two participants died from GC in the BEA group, while none died in the TEA group (Table 4).

## Discussion

To our knowledge, no previous RCT in China has compared participation and preliminary screening effectiveness between biomarker-based screening and traditional endoscopic screening for GC. The endoscopic screening involved 11,358 individuals, yielding a 26.9% participation rate. The TEA group comprised 5,798 participants, while the BEA group had 5,158 participants. In the BEA group, the high-risk rate based on combined questionnaire and biomarker detection was 15.2%, lower than the TEA group (38.9%) which relied solely on questionnaires. Participation rate for endoscopy in the BEA group was higher for high-risk individuals. The detection rate of GC abnormalities did not significantly differ between the TEA and BEA groups. Factors associated with participation rates differed between TEA and BEA, including age, household income, smoking frequency, dyspepsia, digestive tract symptoms, progressive emaciation (in TEA), and education, frequent drinking, frequent consumption of hot, rough and hard food, family history of GC, and history of reflux esophagitis or gastropathy (in BEA). Age group, sex and regular consumption of meat, eggs and milk products were associated with stomach abnormalities. Intention-to-screen and pre-protocol analyses demonstrated no statistically significant discrepancies in cumulative incidence and specific death rates.

**Figure 3:**
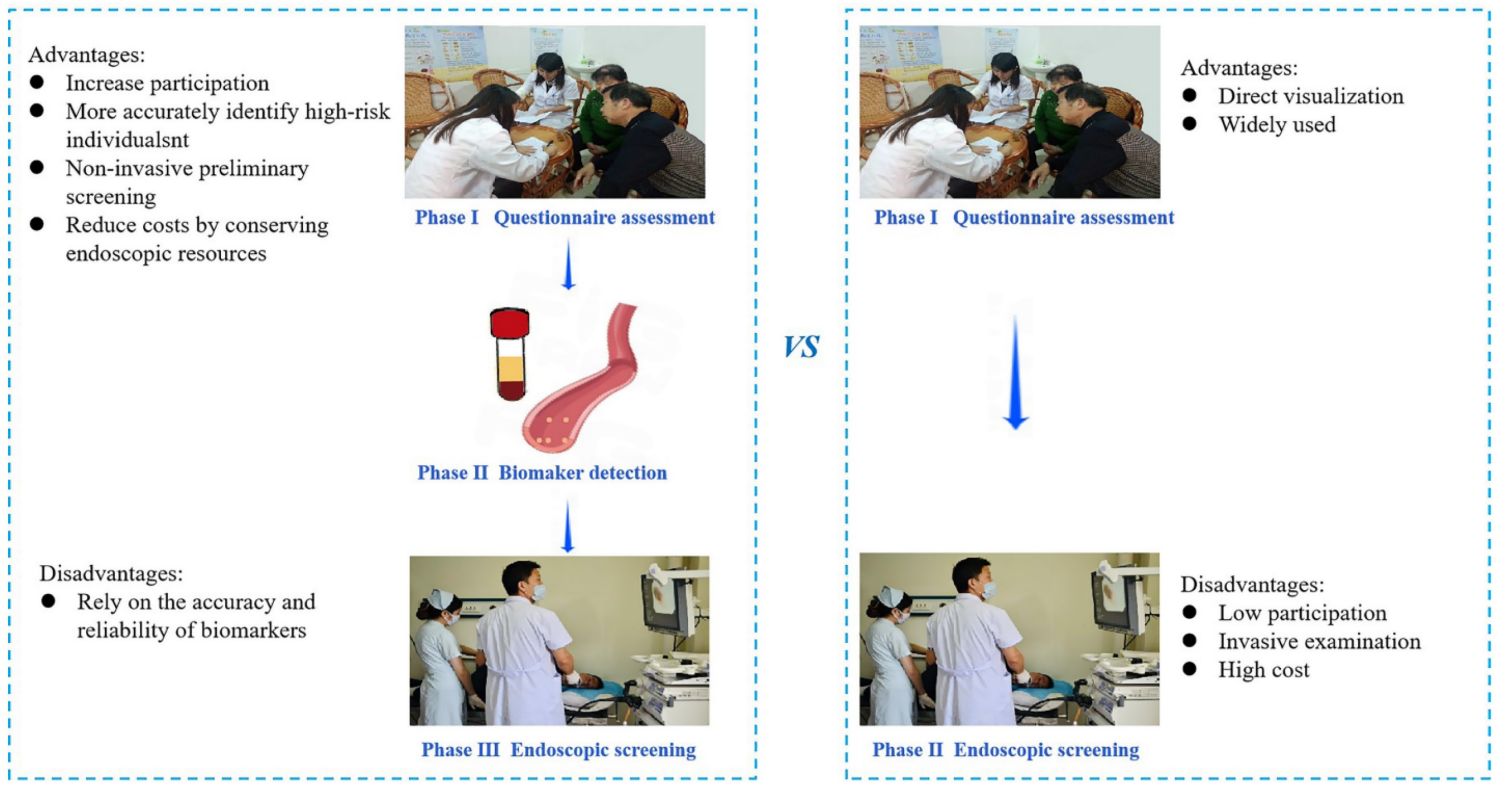
The advantages and disadvantages of two screening methods.

The effects of endoscopic screening on mitigating the occurrence and fatality of GC have been widely studied and verified 3-6. Our findings indicated that the detection rate of baseline screening and cumulative incidence of GC was not significantly different between the TEA and BEA. Therefore, we assumed that the detection efficacy of biomarker-based screening was comparable to traditional endoscopic screening. However, as a new screening method, biomarker-based screening offers distinct advantages in GC screening. First, biomarker-based screening excels in targeting high-risk individuals, which is crucial for optimizing cancer screening efficacy 15. Accurate identification of high-risk populations among healthy individuals reduces anxiety and stress associated with false-positive results for GC 16. The pursuit of diverse methodologies to enhance the precision of high-risk group identification is a focal point for GC screening experts. Traditional high-risk evaluation methods, such as questionnaire surveys extensively studied by epidemiology experts 13, lack the precision achieved by blood biomarker assessment for disease indications. Therefore, it is imperative to explore serum markers to enhance the precision of risk assessments. Our study investigated the combination of a questionnaire survey with five blood biomarkers, with high-risk status assigned only when both assessments were positive. When there was no distinction between screening detection and cumulative incidence between the two arms, biomarker-based screening demonstrated superior precision, as evidenced by a lower high-risk rate and a smaller high-risk population compared to traditional methods. Secondly, biomarker-based screening methods reduce costs by conserving endoscopic resources, including equipment, human resources, and consumables, compared to traditional upper gastrointestinal endoscopy. This is especially notable in terms of human resources, as experienced doctors and technicians are necessary for endoscopy but not for serum biomarker testing. Our findings indicate a lower participation rate of endoscopy in the biomarker-based screening arm, demonstrating reduced use of endoscopic resources without affecting screening detection rates or cumulative mortality compared to the traditional screening arm. Additionally, in the biomarker-based arm, the endoscopy participation rate was higher among high-risk individuals, addressing a key factor in the effectiveness of GC screening—compliance 17. Therefore, improving endoscopic screening compliance by enhancing screening awareness among high-risk participants is essential. In this study, the higher endoscopy participation in the BEA for high-risk individuals suggests that objective biomarker indicators enhance the acceptance of endoscopic screening. The advantages and disadvantages are listed in Figure 3.

In multivariate logistic analysis found that age group, sex and regular consumption of meat, eggs and milk products were significant factors influencing gastric abnormalities. In high risk participants, the risk of gastric abnormalities with age, and male gender had higher risk than female, which were consistent with our previous study 13. For meat, eggs and milk products, it is readily apparent that meat, egg, and milk foods, being rich in high-quality proteins, offer crucial nutrients for the human body, facilitate the repair of gastric mucosa, bolster immunity, enhance digestive function, and thus, exert a protective influence on stomach health.

A multivariate logistic analysis of factors influencing endoscopy compliance revealed that higher educational levels in the BEA were associated with greater adherence to endoscopic screening 18,19, as individuals with higher education levels were more likely to understand the meaning of high cancer risk. Frequent drinking 5 and a history of reflux esophagitis or gastropathy 20 were associated with a higher participation rate, consistent with the results of previous studies 5,20. This may be because of awareness of these unhealthy lifestyles and the history of GC, which prompts individuals to prioritize their health and actively seek opportunities for cancer screening. Conversely, individuals frequently consuming hot, rough and hard food and those with a family history of GC exhibited lower participation rates as they may not have a deeper understanding of cancer screening because they do not know that poor eating habits may harm their health. Individuals with a family history of GC may know that they are a high-risk population and usually voluntarily undergo regular physical examinations, resulting in relatively low compliance with free screening. This is the opposite of Li's results 20. For the TEA, age and household income were positively correlated with the participation rate, indicating that older individuals with a higher income exhibited heightened awareness concerning endoscopic screening. This finding is consistent with previous compliance-related results 20-23. Dyspepsia, digestive tract symptoms, and progressive emaciation are associated with higher participation rates. These diseases require endoscopic examination for diagnosis, and doctors recommend regular follow-up endoscopy 5. Smoking is a significant risk factor for upper gastrointestinal malignancies 24, but participants who smoke frequently but are reluctant to undergo endoscopic screening may believe that smoking is more strongly associated with respiratory tumors. Another probable reason is the lower health literacy. Therefore, integrating these influencing factors in the future can enhance science popularization efforts, address diverse high-risk populations, and improve screening compliance.

This study has several strengths. This study has multiple strengths. One notable strength is its pioneering comparison of conventional endoscopic and biomarker-based screening methods in a community-based cluster randomized controlled trial for GC in China. The involvement of an expert team from the National Cancer Centre ensures the high quality and accuracy of the research design, implementation, and quality control. Additionally, the two-center study design enhances the reliability and robustness of the research findings. However, the limitations of this study warrant careful consideration. Firstly, the short duration of the GC screening assessment (<4 year) could have impacted the outcomes, highlighting the need for longer follow-up in future studies. Secondly, complex screening procedures may affect patient compliance, especially in the BEA where participants must make two hospital visits, one for biomarker testing and another for endoscopy. Biomarker testing also involves delays for feedback before undergoing endoscopy, highlighting the need for process optimization to improve compliance. Third, we encountered limitations in integrating data from the other three non-high-incidence areas of the national study, which resulted in our sample not meeting the original assumptions of the national study. But in terms of baseline detection rates and compliance, we can get effectiveness of biomarker screening modalities.

## Conclusions

We demonstrated that the BEA exhibits several advantages over the TEA, including efficient identification of high-risk individuals for GC, conservation of endoscopic screening resources, and higher compliance with endoscopic screenings. Consequently, we recommend biomarker detection as a secondary screening procedure following questionnaire evaluation to enhance overall efficiency. These findings are valuable for informing national policymaking regarding the screening strategy for GC.

## Figures and Tables

**Table 1:** High risk rate of questionnaire risk assessment on two different arms on demographic characteristic variables.

Factors	Biomarker-based screening arm					Traditional Screening arm			
	Participants (n=5,158)	High-risk participants (n=2,006)	High risk rate (38.9)	*P* value		Participants (n=5,798)	High-risk participants (n=2,307)	High risk rate (39.8)	*P* value
Sex									
Male	1,983 (38.4)	765 (38.1)	38.6	0.738		2,418 (41.7)	924 (40.1)	38.2	0.041
Female	3,175 (61.6)	1,241 (61.9)	39.1			3,380 (58.3)	1,383 (59.9)	40.9	
Age (Years)									
40-49	892 (17.3)	358 (17.8)	40.1	0.005		1,040 (17.9)	449 (19.5)	43.2	0.000
50-59	1917 (37.1)	790 (39.4)	41.2			1,997 (34.4)	899 (39.0)	45.0	
60-69	2,349 (45.6)	858 (42.8)	36.5			2,761 (47.6)	959 (41.6)	34.7	
Body mass index (kg/m^2^)									
<18.5	81 (1.6)	46 (2.3)	56.8	0.000		99 (1.7)	54 (2.3)	54.5	0.003
18.5-24.9	3,769 (73.1)	1,423 (70.9)	37.8			4,328 (74.6)	1,736 (75.2)	40.1	
≥25.0	1,308 (25.3)	537 (26.8)	41.1			1,371 (23.6)	517 (22.4)	37.7	
Education									
Primary school and below	1,205 (23.4)	448 (22.3)	37.2	0.245		1,099 (19.0)	392 (17.0)	35.7	0.007
Middle school	2,842 (55.1)	1,133 (56.5)	39.9			3,661 (63.1)	1,486 (64.4)	40.6	
College and above	1,111 (21.5)	425 (21.2)	38.3			1,038 (17.9)	429 (18.6)	41.3	
Marital status									
Married	4,958 (96.1)	1,936 (96.5)	39.0	0.281		5,560 (95.9)	2,212 (95.9)	39.8	1.000
Unmarried	200 (3.9)	70 (3.5)	35.0			238 (4.1)	95 (4.1)	39.9	
Household income ( CNY)									
≤40000	1,182 (22.9)	489 (24.4)	41.4	0.003		924 (15.9)	386 (16.7)	41.8	0.045
40000-80000	1,804 (35.0)	646 (32.2)	35.8			2,097 (36.2)	791 (34.3)	37.7	
>80000	2,172 (42.1)	871 (43.4)	40.1			2,777 (47.9)	1,130 (49.0)	40.7	
Family size									
<3	1,423 (27.6)	565 (28.2)	39.7	0.479		1,623 (28.0)	605 (26.2)	37.3	0.016
≥3	3,735 (72.4)	1,441 (71.8)	38.6			4,175 (72.0)	1,702 (73.8)	40.8	

**Table 2:** The screening compliance of two different arms on baseline characteristics.

Factors			Biomarker-based screening arm						Traditional Screening arm			*P* value ^a^
	Biomarker detection (n=1,379)					Endoscopy (n=509)			Endoscopy (n=1,222)			
	Participants	Compliance		Positive		Participants	Compliance	*P*	Participants	Compliance	*P*	
**Demographic characteristics**												
Sex												
Male	519 (37.6)	67.8		295 (56.8)		192 (37.7)	65.1	1.000	480 (39.3)	51.9	0.447	0.000
Female	860 (62.4)	69.3		489 (56.9)		317 (62.3)	64.8		742 (60.7)	53.7		0.000
Age (Years)												
40-49	242 (17.5)	67.6		126 (52.1)		90 (17.7)	71.4	0.098	255 (20.9)	56.8	0.000	0.004
50-59	556 (40.3)	70.4		302 (54.3)		184 (36.1)	60.9		509 (41.7)	56.6		0.214
60-69	581 (42.1)	67.7		356 (61.3)		235 (46.2)	66		458 (37.5)	47.8		0.000
Body mass index (kg/m^2^)												
<18.5	34 (2.5)	73.9		19 (55.9)		17 (3.3)	89.5	0.074	27 (2.2)	50	0.282	0.000
18.5-24.9	973 (70.6)	68.4		543 (55.8)		348 (68.4)	64.1		936 (76.6)	53.9		0.000
≥25.0	372 (27.0)	69.3		222 (59.7)		144 (28.3)	64.9		259 (21.2)	50.1		0.000
Education												
Primary school and below	301 (21.8)	67.2		177 (58.8)		110 (21.6)	62.1	0.012	172 (14.1)	38.4	0.000	0.000
Middle school	771 (55.9)	68		457 (59.3)		286 (56.2)	62.6		817 (66.9)	72.1		0.000
College and above	307 (22.3)	72.2		150 (48.9)		113 (22.2)	75.3		233 (19.1)	54.8		0.000
Marriage												
Married	1327 (96.2)	68.5		753 (56.7)		488 (95.9)	64.8	0.886	1181 (96.6)	53.4	0.064	0.000
Unmarried	52 (3.8)	74.3		31 (59.6)		21 (4.1)	67.7		41 (3.4)	43.2		0.030
Household income (CNY)												
≤40000	314 (22.8)	64.2		189 (60.2)		133 (26.1)	70.4	0.193	194 (15.9)	50.3	0.040	0.000
40000-80000	428 (31.0)	66.3		248 (57.9)		158 (31)	63.7		399 (32.7)	50.4		0.000
>80000	637 (46.2)	73.1		347 (54.5)		218 (42.8)	62.8		629 (51.5)	55.7		0.022
Family size												
<3	398 (28.9)	70.4		234 (58.8)		157 (30.8)	67.1	0.454	322 (26.4)	53.2	0.922	0.000
≥3	981 (71.1)	68.1		550 (56.1)		352 (69.2)	64		900 (73.6)	52.9		0.000
**Potential risk factors**												
Smoking frequently												
Yes	74 (5.4)	64.3		47 (63.5)		27 (5.3)	57.4	0.342	48 (3.9)	34.8	0.000	0.010
No	1305 (94.6)	69		737 (56.5)		482 (94.7)	65.4		1174 (96.1)	54.1		0.000
Drinking frequently												
Yes	224 (16.2)	70.9		113 (50.4)		82 (16.1)	72.6	0.083	178 (14.6)	50.9	0.423	0.000
No	1,155 (83.8)	68.3		671 (58.1)		427 (83.9)	63.6		1,044 (85.4)	53.3		0.000
Eating mildewed, fermented and high-salt containing foods frequently												
Yes	511 (37.1)	68.3		301 (58.9)		188 (36.9)	62.5	0.287	535 (43.8)	52.3	0.624	0.002
No	868 (62.9)	69		483 (55.6)		321 (63.1)	66.5		687 (56.2)	53.5		0.000
Eating hot and rough food frequently												
Yes	596 (43.2)	69.1		355 (59.6)		210 (41.3)	59.2	0.003	539 (44.1)	51.9	0.387	0.022
No	783 (56.8)	68.5		429 (54.8)		299 (58.7)	69.7		683 (55.9)	53.8		0.000
History of reflux esophagitis or gastropathy												
Yes	119 (8.6)	71.3		77 (64.7)		57 (11.2)	74.0	0.102	101 (8.3)	57.4	0.253	0.018
No	1,260 (91.4)	68.5		707 (56.1)		452 (88.8)	63.9		1,121 (91.7)	52.6		0.000
Family history of upper gastrointestinal cancer												
Yes	498 (36.1)	73.2		267 (53.6)		157 (30.8)	58.8	0.012	323 (26.4)	53	1.000	0.127
No	881 (63.9)	66.4		517 (58.7)		352 (69.2)	68.1		899 (73.6)	53		0.000
Dysphagia												
Yes	212 (15.4)	68.2		125 (59)		84 (16.5)	67.2	0.632	240 (19.6)	70.0	0.000	0.645
No	1,167 (84.6)	68.8		659 (56.5)		425 (83.5)	64.5		982 (80.4)	50.0		0.000
Swallowing pain (sternum or back)												
Yes	179 (13.0)	64.4		107 (59.8)		72 (14.1)	67.3	0.658	121 (9.9)	65.8	0.000	0.891
No	1,200 (87.0)	69.4		677 (56.4)		437 (85.9)	64.5		1,101 (90.1)	51.9		0.000
Digestive tract symptom (loss of appetite, nausea and vomiting, etc.)												
Yes	1,110 (80.5)	70.3		646 (58.2)		425 (83.5)	65.8	0.317	1,044 (85.4)	57.8	0.000	0.000
No	269 (19.5)	63		138 (51.3)		84 (16.5)	60.9		178 (14.6)	35.6		0.000
Black stool												
Yes	75 (5.4)	67.6		42 (56)		32 (6.3)	76.2	0.160	127 (10.4)	61.4	0.014	0.099
No	1,304 (94.6)	68.8		742 (56.9)		477 (93.7)	64.3		1,095 (89.6)	52.1		0.000
Progressive emaciation												
Yes	51 (3.7)	76.1		31 (60.8)		22 (4.3)	71	0.598	51 (4.2)	77.3	0.000	0.675
No	1,328 (96.3)	68.5		753 (56.7)		487 (95.7)	64.7		1,171 (95.8)	52.3		0.000
												

^a^ Indicates comparison of adherence to screening between the two study groups.

**Table 3:** Multivariate logistic analysis of influence factors in two groups.

Variables	Biomarker-based screening arm							Traditional Screening arm	
	Biomarker detection				Endoscopy			Endoscopy	
	Adjusted OR (95%CI)		*P*		Adjusted OR (95%CI)	*P*		Adjusted OR (95%CI)	*P*
Age (Years)									
40-49	—		—		—	—		1.290(1.019-1.632)	0.034
50-59	—		—		—	—		1.362(1.126-1.646)	0.001
60-69	—		—		—	—		Ref	
Education									
Primary school and below	—		—		Ref			—	—
Middle school	—		—		0.990(0.688-1.425)	0.956		—	—
College and above	—		—		1.734(1.066-2.823)	0.027		—	—
Household income ( CNY )									
≤40000	Ref				—	—		Ref	
40000- 80000	1.087(0.847-1.396)		0.511		—	—		1.372(1.073-1.754)	0.012
>80000	1.607(1.257-2.053)		0.000		—	—		1.199(0.926-1.552)	0.168
Family size									
<3	1.325(1.056-1.661)		0.015		—	—		—	—
≥3	Ref				—	—		—	—
Smoking frequently									
Yes	—		—		—	—		0.450(0.304-0.665)	0.000
No	—		—		—	—		Ref	
Drinking frequently									
Yes		—	—		1.616(1.029-2.540)	0.037		—	—
No		—	—		Ref			—	—
Eating hot, rough and hard food frequently									
Yes		—	—		0.628 (0.462-0.853)	0.003		—	—
No		—	—		Ref			—	—
Dysphagia									
Yes		—	—		—	—		2.256(1.745-2.916)	0.000
No		—	—		—	—		Ref	
Family history of upper gastrointestinal cancer									
Yes	1.456(1.178-1.800)		0.001		0.720(0.523-0.989)	0.022		—	—
No	Ref				Ref			—	—
Digestive tract symptom									
Yes	1.558(1.235-1.966)		0.000		—	—		2.340(1.893-2.892)	0.000
No	Ref				—	—		Ref	
History of reflux esophagitis or gastropathy									
Yes	—		—		1.892(1.099-3.260)	0.043		—	—
No	—		—		Ref			—	—
Progressive emaciation									
Yes	—		—		—	—		2.850(1.542-5.266)	0.001
No	—		—		—	—		Ref	
									

**Table 4:** The risk of GC incidence and mortality in intention-to-screen and per-protocol analyses.

		Intention-to-screen analyses	Per-protocol analyses
GC incidence	**Tradional screening arm**		
	GC cases	13	3
	**Biomarker-based screening arm**		
	GC cases	18	6
	Hazard Ratio (95%CI)	1.99(0.96-4.13)	2.29(0.57-9.17)
	*P* value	0.066	0.241
GC mortality	**Tradional screening arm**		
	Death case with GC	4	0
	**Biomarker-based screening arm**		
	Death case with GC	7	2
	Hazard Ratio (95%CI)	3.03(0.87-10.64)	-
	*P* value	0.083	-
